# Effects of an Oral Contraceptive on Dynamic Brain States and Network Modularity in a Serial Single-Subject Study

**DOI:** 10.3389/fnins.2022.855582

**Published:** 2022-06-14

**Authors:** Kristian Høj Reveles Jensen, Drummond E-Wen McCulloch, Anders Stevnhoved Olsen, Silvia Elisabetta Portis Bruzzone, Søren Vinther Larsen, Patrick MacDonald Fisher, Vibe Gedsoe Frokjaer

**Affiliations:** ^1^Neurobiology Research Unit, Copenhagen University Hospital Rigshospitalet, Copenhagen, Denmark; ^2^Psychiatric Center Copenhagen, Copenhagen, Denmark; ^3^Faculty of Health and Medical Sciences, University of Copenhagen, Copenhagen, Denmark; ^4^Department of Applied Mathematics and Computer Science, DTU Compute, Kongens Lyngby, Denmark

**Keywords:** oral contraceptive (OC), functional connectivity (FC), functional magnetic resonance imaging (fMRI), menstrual cycle, steroid hormones, dynamic functional connectivity (dFC), hormonal contraceptive, brain modularity

## Abstract

Hormonal contraceptive drugs are used by adolescent and adult women worldwide. Increasing evidence from human neuroimaging research indicates that oral contraceptives can alter regional functional brain connectivity and brain chemistry. However, questions remain regarding static whole-brain and dynamic network-wise functional connectivity changes. A healthy woman (23 years old) was scanned every day over 30 consecutive days during a naturally occurring menstrual cycle and again a year later while using a combined hormonal contraceptive. Here we calculated graph theory-derived, whole-brain, network-level measures (modularity and system segregation) and global brain connectivity (characteristic path length) as well as dynamic functional brain connectivity using Leading Eigenvector Dynamic Analysis and diametrical clustering. These metrics were calculated for each scan session during the serial sampling periods to compare metrics between the subject’s natural and contraceptive cycles. Modularity, system segregation, and characteristic path length were statistically significantly higher across the natural compared to contraceptive cycle scans. We also observed a shift in the prevalence of two discrete brain states when using the contraceptive. Our results suggest a more network-structured brain connectivity architecture during the natural cycle, whereas oral contraceptive use is associated with a generally increased connectivity structure evidenced by lower characteristic path length. The results of this repeated, single-subject analysis allude to the possible effects of oral contraceptives on brain-wide connectivity, which should be evaluated in a cohort to resolve the extent to which these effects generalize across the population and the possible impact of a year-long period between conditions.

## Introduction

Naturally cycling women undergo menstrual cycles for approximately a third of their lifespan, involving profound sex steroids level fluctuations across 24- to 36-day cycles, frequently with coinciding fluctuations in mood, impulsivity, and irritability (Pletzer et al., 2017; Lewis et al., 2019). Such hormonal rhythms are significantly altered by oral contraceptive (OC) medication, which (Iversen et al., 2020) are used by more than 100 million women globally (Brynhildsen, 2014) and in Denmark 42% of women in the reproductive age use OCs, while 80% have used them at some point in their lives (Skovlund et al., 2016). The most common OCs combine an estrogen and a progestin to downregulate endogenous ovarian sex steroid hormone levels, resulting in inhibited follicular growth, egg maturation, and ovulation, thus preventing pregnancy. There has been a significant focus on somatic side effects of OC use, such as the increased risk of thromboembolic disease (Amoozegar et al., 2015; Roach et al., 2015; Keenan et al., 2018). However, women also report adverse impacts on psychological wellbeing, e.g., mood instability, irritability, sadness, symptoms of depression and anxiety, and a decrease or lack of libido (Guen et al., 2021) which has recently received increased attention. Epidemiological studies show an association between starting an OC and the emergence of depressive episodes, especially among adolescents (Skovlund et al., 2016; Zettermark et al., 2018; Anderl et al., 2020, 2021).

Sex-steroid milieu changes have been found to alter brain biology, including hippocampal plasticity (Barth et al., 2016; Taylor et al., 2020) and serotonergic neurotransmission (Barth et al., 2015), both crucial to maintaining mental health (Frokjaer, 2020). In addition, mental disorders, such as anxiety and mood disorders, can be exacerbated during certain menstrual cycle phases, including premenstrual symptom worsening (Pinkerton et al., 2010; Green and Graham, 2022; Kuehner and Nayman, 2021). Hormonal fluctuations across the menstrual cycle putatively trigger severe depressive symptoms in some women, i.e., premenstrual dysphoric disorder (PMDD), which can be treated with specific OCs but worsened by others (Rapkin et al., 2019).

Although OCs are beneficial for reproductive health and well tolerated by some women, it is necessary that we examine their effect on brain function and how this may affect mental health. Previous neuroimaging studies of the relationship between hormonal dynamics during the menstrual cycle, OC, and brain function have predominately used task-based functional magnetic resonance imaging (fMRI) during a few selected time points, i.e., during the follicular and luteal phases (Dubol et al., 2020). Most studies on OCs use a between-subject design that can be subject to individual-based confounding factors, e.g., duration and onset of OC use (Montoya and Bos, 2017) and self-selection bias (Brønnick et al., 2020). Test-retest reliability of task-based fMRI measures is overall poor and is further impacted by repeated measurements, i.e., habituation effects (Elliott et al., 2020). Thus, studies have increasingly investigated resting-state fMRI (rs-fMRI) by applying dense sampling across multiple time points in individual subjects (Arélin et al., 2015; Pritschet et al., 2021b).

Recently, the “28andMe” project (Pritschet et al., 2020) acquired serial measures of rs-fMRI of one woman once per day during one natural menstrual cycle. A year later, the study was repeated while on a combined OC with estrogen and an androgenic progestin. The initial report on this data found that 17β-oestradiol appeared to facilitate tighter coherence within static functional brain-networks, while progesterone had the opposite effect (Pritschet et al., 2020). Additionally, network reorganization occurred in several networks across the menstrual cycle, most strikingly in a default mode subnetwork localized to prefrontal cortex regions during the ovulatory hormone peaks (Mueller et al., 2021). This reorganization was not present while on OC, despite a similar mid-cycle oestradiol peak on OC, suggesting that this OC constrained or blunted default mode network (DMN) connectivity during estrogen fluctuations.

These studies did not evaluate graph-theoretical estimates of overall brain connectivity either on a network-wise or global level. Modularity and system segregation are static estimates of whole-brain connectivity related to relative within- and between-network connectivity strength (Cohen and D’Esposito, 2016; Sporns and Betzel, 2016). In contrast, characteristic path length is a network-independent measure of “connectedness” between brain regions, representing an approximation of capacity for information flow throughout the brain (Rubinov and Sporns, 2011). In contrast to “static” functional connectivity measures, which evaluate average connectivity across the entire scan session, thus presuming signal stationarity, dynamical functional connectivity (dFC) provides a framework for estimating signal fluctuations within a resting-state scan session (Chang and Glover, 2009; Preti et al., 2016; Cabral et al., 2017). Using dFC, we may characterize discrete time-varying brain connectivity patterns, denoted brain states, whose expression may be related to the use of OC medication.

Here we leverage the repeated rs-fMRI acquisition framework of the “28andMe” project, where we evaluate whether the above graph-theoretical and dynamical whole-brain connectivity measures differed throughout the natural and OC cycles measured one year apart. This explorative approach characterizes how these measures may vary during this subject’s natural cycle and when on OC medication. Evaluating differences in these measures during the natural and OC cycle offers a novel perspective into how OC use may affect brain function. The nature of the dataset does not allow us to discriminate with certainty between effects of OC or effects of time between the two series of measurements, which should be kept in mind when interpreting the results, but offers a fundament for hypotheses to be tested in other study designs.

## Materials and Methods

The 28andMe dataset for this study was obtained from the OpenNeuro database (Accession Number: ds002674, version 1.0.5, doi: 10.18112/openneuro.ds002674.v1.0.5) (Pritschet et al., 2021a) and is available under the CC0 license. The subject, the study design, and magnetic resonance imaging (MRI) acquisition are described previously (Pritschet et al., 2020, 2021b; Taylor et al., 2020) and summarized here. MRI acquisition and fMRI preprocessing steps are described in the Supplementary Material.

### The Subject and Study Design

The subject was a right-handed 23-year-old Caucasian female graduate student with no neuropsychiatric or endocrine disorders or prior head trauma history. She had a history of regular menstrual cycles (no missed periods, cycle length 26–28 days) and had not taken hormone-based medication in the 12 months before study onset.

The subject underwent rs-fMRI daily at 11 a.m. for 30 consecutive days. 12 months later the subject repeated the 30-day fMRI protocol while on a monophasic hormonal contraceptive regime of 21 active days (20 μg ethinylestradiol and 100 μg levonorgestrel, Aubra, Afaxys Pharmaceuticals) and 7 placebo days, which she began 10 months before the second data collection. The subject began each test session with daily behavioral assessments and blood measurements, see Taylor et al. (2020).

**FIGURE 1:**
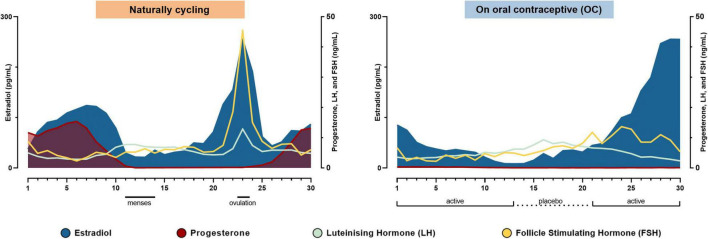
Hormonal timelines in each of the two conditions. The subject’s hormone levels during the natural cycle and while taking the oral contraceptive in the order of experiment days. Modified from Taylor et al. (2020).

Hormone levels were not attached in the OpenNeuro data set and were extracted from Figure 1 from Taylor et al. (2020) using WebPlotDigitizer 4.5 (Rohatgi, 2021). The study days were transformed into cycle days as in Taylor et al. (2020) and plotted in Figure 1.

### Graph-Theory Measures

To evaluate graph-theory measures, we considered a graphical representation of the brain, where brain regions are described as nodes and edges between those nodes that represent connection strength. The set of connections (i.e., connectivity strengths) can be represented as a connectivity matrix, a diagonally symmetrical *n* × *n* matrix containing continuous values, where *n* is the number of nodes (Schaefer et al., 2018) and each matrix element represents the magnitude of estimated static connectivity between that node pair, expressed either as a Pearson’s correlation coefficient or Fisher’s transformed r-to-z (r2z) score.

We summarize the graphical representation of static brain connectivity using several established metrics (Rubinov and Sporns, 2009) including modularity (Cohen and D’Esposito, 2016) and system segregation (Chan et al., 2014) which relate to network connectivity through the lens of modular brain networks, and characteristic path length (CPL) (Rubinov and Sporns, 2009), which evaluates connectivity of the brain considered as a whole. See Supplementary Figure 1A for a simplified diagram. Brain regions were allocated based on the Schaefer-400 atlas (Schaefer et al., 2018). This matrix can be expressed as a graph that can be made sparse by applying a threshold and retaining only edges above that threshold. Depending on the metric, edge strength can be weighted, reflecting observed connectivity strength (a value between 0 and 1), or binarized (0 or 1) based on the defined threshold. In the absence of an *a priori* optimal threshold, binarization was performed over a range of thresholds. For modularity and system segregation analyses, regions were allocated to seven canonical resting-state networks: the visual, somatomotor, dorsal attention, ventral attention (salience), limbic, default mode, and executive control networks from a commonly used parcelation (Yeo et al., 2011). Further elaboration on the precise graph theory metrics utilized in this analysis are available in the Supplementary Material.

### Dynamic Functional Brain Connectivity

We estimate dynamic connectivity structures using Leading Eigenvector Dynamics Analysis (LEiDA) (Cabral et al., 2017), followed by diametrical clustering as in Olsen et al. (2021; see Supplementary Figures 1B–E). The brain was parcelated into cortical regions from the Schaefer-100 atlas (Schaefer et al., 2018). LEiDA assesses dynamic functional connectivity by estimating regional instantaneous phases through the Hilbert transform, constructing a *P*×*P* phase coherence map for every timepoint *t*, and extracting its leading eigenvector (see Supplementary Section 2 and Supplementary Figure 1 for more details). The leading eigenvector represents the dominant instantaneous connectivity pattern for every functional volume acquired. Clusters in the pool of leading eigenvectors (assumed independent) are informative of general states of brain connectivity. Diametrical clustering is a *k*-means type clustering algorithm which acknowledges the unit norm and sign invariance of eigenvectors and which, for a specified number of states *k* produces dynamic brain connectivity states and a labeling for all acquired volumes (Dhillon et al., 2003). We evaluated *k* in the range 2–20 and, for each scan session and *k*, computed the fractional occurrence of all states.

### Statistical Analysis

We evaluated differences in brain imaging measures between the natural cycle and OC condition using paired *t*-tests, our statistical significance threshold was set at α < 0.05. As our estimates across both 30-day periods are effectively time series, we considered evidence for autocorrelation. See Supplementary Figure 2 for correlograms of the paired difference for all metrics. Based on visual inspection we observed potential autoregressive effects for bCPL and modularity. For these, we modeled the autocorrelation with lag 1 using a generalized least squares regression of the paired differences using the *nlme* package version 3.1 for R and reported the associated *p*-value (Pinheiro et al., 2021). One ROI (122/400 from Schaefer 400, temporal pole) was removed from bCPL analyses as it produced infinite path lengths in some scans; this had a negligible impact on other bCPL values.

All confidence intervals (*CI*) presented are 95% confidence intervals. For tests of brain state fractional occurrence, we employ within-*k*, Bonferroni-corrected statistical significance thresholds to control the family-wise error rate (FWER).

### Visualizations and Code

MATLAB (The MathWorks Inc.) and R 4.1^1^ were used to generate the results presented here; the corresponding code has been made publicly available at https://github.com/anders-s-olsen/28andme. BrainNet Viewer 1.7^2^ was used to generate connectivity visualizations (Xia et al., 2013). Some plots were constructed using *ggplot2* in R (Wickham, 2016). The BrainConnectivity toolbox was used to estimate CPLs, and bCPL was calculated with the *distance_bin* function (Rubinov and Sporns, 2009).

## Results

### Weighted Graph-Theory Measures

#### System Segregation

**FIGURE 2:**
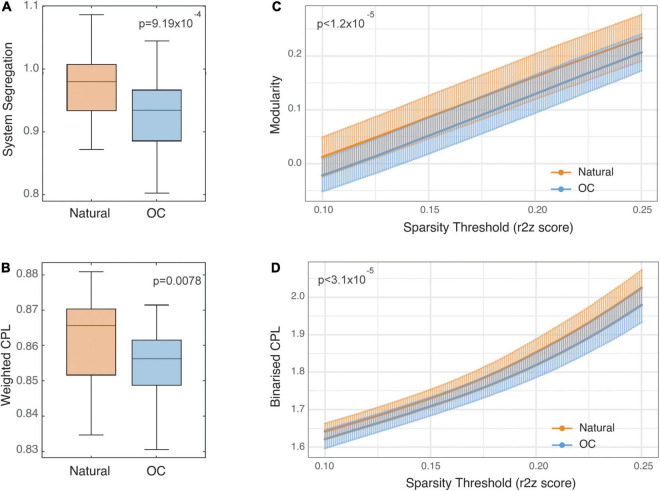
Static functional connectivity comparisons between naturally cycling and oral contraceptive (OC) condition scans. Panels **(A,B)** show Tukey’s boxplots representing system segregation and weighted characteristic path length values (CPL), respectively. The horizontal lines represent medians, and the vertical limits of the colored area represent the first and third quartiles. Notches represent 95% confidence intervals for comparing medians, calculated as $\frac{1.58\times \mathrm{IQR}}{\sqrt{n}}$. Panels **(C,D)** show modularity and binarized CPL values reported for each condition at a range of sparsity threshold values between 0.1 and 0.25 and are plotted as mean ± SD.

System segregation across the natural cycle (0.98 ± 0.05) was statistically significantly greater than across the OC cycle (0.92 ± 0.06; difference: 0.052, 95% *CI*: 0.023:0.081, *p* = 9.19 × 10^–4^; Figure 2A) with a large effect size (Cohen’s *d* = 0.97, 95% *CI*: 0.33–1.61).

#### Weighted Characteristic Path Length

Weighted characteristic path length across the natural cycle (0.86 ± 0.01) was statistically significantly greater than across the OC cycle (0.85 ± 0.01; difference: 0.0071, 95% *CI*: 0.002–0.012, *p* = 0.0078; Figure 2B) with a medium effect size (*d* = 0.62, 95% *CI*: 0.15–1.11).

### Binarized Graph-Theory Measures

#### Modularity

Estimates of modularity were statistically significantly higher during the natural cycle (0.01 at threshold 0.1, 0.23 at threshold 0.25) compared to the OC cycle (−0.02 at threshold 0.1, 0.20 at threshold 0.25) across the range of connectivity thresholds (range of mean difference in modularity 0.028–0.036; all *p* ≤ 1.2 × 10^–5^; Figure 2C) with a large to medium effect size across the range of connectivity thresholds (*d* = 1.00, 95% *CI*: 0.46–1.55 at threshold 0.1 to *d* = 0.69, 95% *CI*: 0.26–1.11 at threshold 0.25). See Supplementary Figure 3 for plots of estimates and *p*-values across threshold values.

#### Binarized Characteristic Path Length

Estimates of bCPL were statistically significantly longer in the natural cycle (1.64 at threshold 0.1, 2.02 at threshold 0.25) compared to the OC cycle (1.62 at threshold 0.1, 1.98 at threshold 0.25) across the range of connectivity thresholds (range of mean difference in bCPL 0.021–0.049; all *p* ≤ 3.1 × 10^–5^; Figure 2D) with a large effect size across the range of connectivity thresholds (*d* = 0.93, 95% *CI*: 0.29–1.56 at threshold 0.1 to *d* = 1.02, 95% *CI*: 0.33–1.70 at threshold 0.25). See Supplementary Figure 3 for plots of estimates and *p*-values across threshold values.

### Dynamic Functional Brain Connectivity

**FIGURE 3:**
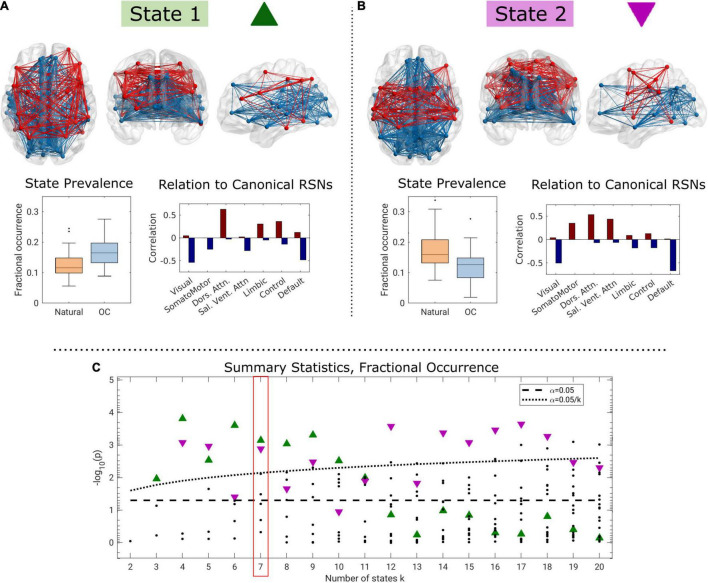
Two brain states for *k* = 7 identified as having significantly elevated fractional occurrence when oral contraceptive medication is used compared to a natural menstrual cycle **(A)**, and opposite **(B)**, and summary *p*-values across the range of *k* **(C)**. In the connectivity visualizations, nodes are only shown if the corresponding centroid element has a strength of at least 50% of the maximum absolute loading. In panel **(C)**, green triangles correspond to state 1, purple triangles to state 2, and the dotted black line corresponds to the within-*k* Bonferroni-corrected threshold for statistical significance.

The evaluation of differences between natural and OC cycle dynamic functional connectivity identified two brain states for which the fractional occurrence differed statistically significantly in 8 and 10 of 19 models, respectively. In total, 209 statistical tests were performed. Summary Bonferroni-corrected *p*-values are presented in Figure 3C across the range of *k*∈{2,…,20}.

For *k*≥3, we observed one brain state (“State 1,” green triangle in Figure 3C) for which the fractional occurrence was significantly higher in the OC state (*k* = 7: estimate = 0.0455; *CI* = 0.0209, 0.0701; *p* < 0.001; *p*_FWER_ = 0.005, Cohen’s *d* = 1.00; Figure 3A). The difference in fractional occurrence of this state between the natural cycle and OC condition was statistically significant over the interval *k* ∈ {3,…,10}. State 1 is mainly characterized by functional coherence between regions related to the dorsal attention network and, to some extent, limbic, and control networks (see Supplementary Figure 4 for centroid loadings). These are in turn antisynchronous with regions related to the visual and DMNs and partly to the salience/ventral attention network (Figure 3A). Brain state 1 was structurally similar across *k* (Supplementary Figure 4).

In contrast, for *k*≥4, we observed a second brain state (“State 2,” purple triangle in Figure 3C) for which the fractional occurrence was statistically significantly higher during the natural cycle (*k* = 7: estimate = −0.0502; 95% *CI* = −0.0789, −0.0213; *p* = 0.001; *p*_FWER_ = 0.009, *d* = 0.92, Figure 3B). This effect was statistically significant for *k* ∈ {4,5,7,9,12,…,18}. We note that the highlighted brain states for *k* ∈ {4,5,9} are structurally somewhat different than for the rest of the range, particularly regarding the representation of the visual network (see Supplementary Figure 4). Brain state 2 is characterized by functional coherence between the dorsal attention and salience networks, and, to some extent, the somatomotor network. These are in turn antisynchronous with regions related to the visual, default-mode, control, and limbic networks (Figure 3 and Supplementary Figure 4).

## Discussion

Here we investigated static and dynamic functional connectivity during two 30-day periods a year apart during which a single healthy woman completed daily rs-fMRI scan sessions. Contrasting the 30-day period during which the subject had her natural menstrual cycle with the data from the OC condition, we observed higher modularity, system segregation, and characteristic path length during her natural cycle relative to OC (Table 1). These findings suggest that OC may alter brain network organization and point to a whole-brain connectivity architecture that is less strongly partitioned into resting-state networks during OC use. Dynamic functional connectivity analysis identified two discrete brain states for which the fractional occurrence was significantly altered between the natural cycle and OC condition. Together, these results suggest an association between OC use and changes in brain network segregation, including connectivity dynamics in this single subject.

**TABLE 1:** Graph theory measures and state prevalence during oral contraceptive (OC) and naturally cycling state 1 and 2 are both states involving the frontoparietal network.

	Modular graph theory		Global graph theory		Leading Eigenvector Dynamics Analysis (LEiDA)	
			
	Modularity	System segregation	Weighted characteristic path length (CPL)	Binarized CPL	Fractional occurrence	
OC vs natural	↓Modularity	↓Segregation	↓Path length	↓Path length	↑State 1 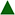	↓State 2 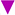

### Brain Connectivity Organization Associated With Oral Contraceptive Use

We show that the subject’s static brain connectivity during rs-fMRI in the OC cycle relative to the natural cycle was less modular and less segregated into independent systems, i.e., had greater between vs. within network connectivity. Also, on average, shorter paths were required for information transfer between brain regions in the OC cycle.

A recent meta-analysis of 12 studies showed that low brain modularity was associated with major depressive disorder (MDD) with a small to medium effect size (Hedge’s *g* = −0.33) (Xu et al., 2021). CPL is a measure of information transfer efficiency (Bullmore and Sporns, 2012). In contrast to modularity, system segregation and CPL were not associated with MDD (Xu et al., 2021), and current data investigating the relation between CPL and MDD and anxiety disorders and the effects of previous pregnancy and PMDD are conflicting (Dan et al., 2020; Chu et al., 2021; Guo et al., 2021; Zhang et al., 2021). Additionally, across MDD patients, these measures appear to vary by the age of disease onset, further complicating interpretation of these metrics (Yun and Kim, 2021).

Transient reorganization of functional brain networks during the NC has been observed in the 28andme dataset by Mueller et al. (2021); the most striking reorganization occurred in a DMN subnetwork localized to regions of the prefrontal cortex, during peaks in oestradiol and gonadotropins, which was not observed during OC despite a similar oestradiol peak. This suggests that the OC-induced suppression of gonadotropins reduces network flexibility and the brain’s ability to reorganize at the mesoscale level in response to oestradiol (Mueller et al., 2021). Similarly, using a novel metric of brain macroscale information processing known as brain turbulence, higher turbulence levels at lower scales (i.e., long distances in the brain) and higher information transmission across scales were observed in the luteal versus the follicular phase (Filippi et al., 2021). In contrast, during OC-use, there were no shifts in information processing across the OC-cycle. Taken together, these findings could be interpreted as increased stability or blunted dynamics by the OC-induced hypothalamic-pituitary-gonadal (HPG) axis suppression. This may be related to the decreased modularity, system segregation and CPL that we observed during OC when compared to NC.

A previous analysis of the 28andMe data showed oestradiol’s ability to modulate information transfer efficiency within the DMN was present in both NC and OC (Pritschet et al., 2020). However, the subject has a retained oestradiol peak during OC, which is uncommon (Río et al., 2018), and may be specific to that subject or that cycle. We cannot exclude that, women on OCs with a more profoundly suppressed HPG axis would display stronger effects of OC use compared to natural cycling, an effect that should be considered in future population studies evaluating similar effects.

### Oral Contraceptive Associated With Changes in Brain Dynamics

The highlighted dFC brain states 1 and 2 were characterized by loadings (Figure 3 and Supplementary Figure 4), that to a large degree, mapped on to functional networks (Yeo et al., 2011). State 1, and the OC condition, is visually characterized by reduced within-network connectivity for the DMN, and slightly increased within-network connectivity for the dorsal attention network. Although this is numerically consistent with previous studies reporting, reduced DMN-connectivity in depressed individuals, it is relevant to note that this effect is of small size and variable across study populations (Yan et al., 2019; Tozzi et al., 2021). Although the DMN and visual networks appear together with the antisynchronous dorsal attention network in both states, it appears that the somatomotor, salience, limbic, and executive control networks shift their associations (Figure 3). However, for both the executive control and limbic networks the effects appear to be confined to single regions rather than the network. Expressed crudely, OC use is associated with a shift in dorsal attention and DMN connectivity with the limbic and executive control networks to the somatomotor and salience networks in the evaluated subject. Functional hyperconnectivity has previously been reported in relation to depression. A similar study in the same dataset employed edge time series (Esfahlani et al., 2020) to detect communities of distinct high-amplitude fluctuations (Greenwell et al., 2021). The study focused on two communities, of which one displayed opposed fluctuation between regions in the DMN with the dorsal, salience, and sensorimotor networks, similar to state 2 presented here. Likewise, the second community was characterized by opposed co-fluctuations in the control and dorsal attention networks with the DMN, similar to state 1 presented here. Although edge time series is fundamentally different to LEiDA and diametrical clustering, it is encouraging that the two studies show converging results. Taken together, our findings suggest hormonal contraceptive effects on the occurrence of brain states and corresponding network-specific connectivity in a single subject.

### Methodological Considerations

The major limitation of this study is that all analyses were performed on a single individual, across single cycles and in relation to one single type of OC, and that data acquisition for the two conditions was performed 1 year apart. Future studies in a cohort of women, ideally across multiple cycles are required to establish population-level effects of OC use on distributed brain connectivity estimates. Additionally, it is unclear whether the observed differences in brain connectivity are due to OC use (or not) or other factors that may have changed between these two scan periods. Nevertheless, it is a strength of these data that this individual was scanned 30 times, limiting sensitivity to spurious differences that may have emerged were data collected across fewer days. This represents an intriguing dataset with which we have generated observations that support novel hypotheses regarding OC effects on brain connectivity that can be evaluated in larger cohorts.

The reported estimates of functional connectivity based on binarized connectivity matrices are limited due to the undirected selection of a sparsity threshold. We demonstrate that reporting results over a range of thresholds that preserve a balance between randomness and regularity of network connectivity (Bassett and Bullmore, 2016) can provide results that are less constrained by *a priori* threshold selection, though further work must work to refine the optimal threshold range for the human functional connectome. Additionally, we show convergence between binarized and weighted analysis frameworks for both global and network-based analyses.

By using LEiDA to delineate dFC structures, we impose little prior knowledge on the optimal number of brain states and instead evaluate a range of brain state partitions. Inevitably, there will be discrepancies between estimated brain states, dependent on model order *k*. For both highlighted brain states, slight alterations in state loadings occur in the transitions from *k* = 5 to *k* = 6, and again from *k* = 11 to *k* = 12 (Supplementary Figure 4B). This indicates that the significant changes in brain state dynamics observed in our statistical tests probably arise from only a subset of the regions in the identified brain states.

Combined OCs with the antiandrogenic progestins, i.e., Drospirenone and Desogestrel compared to androgenic, appear to have different effects on functional brain connectivity, cognition and mood (Poromaa and Segebladh, 2012; Pletzer et al., 2015, 2016). The OC used by the individual in this study was a combined OC with the androgenic progestin Levonorgestrel. Thus, the effects observed in this individual may be particular to this type of OC.

### Perspectives on Future Research

Several epidemiological studies consistently suggest an association between starting an OC and the emergence of depressive episodes, especially among adolescents (Skovlund et al., 2016; Zettermark et al., 2018; Anderl et al., 2020, 2021). Of concern, adolescents are initiating OC use at increasingly younger ages, often shortly after the onset of puberty (Parkes et al., 2009), when the brain is undergoing organizational changes and maturation related to the surge of sex hormones (Levitt, 2003), which may affect brain architecture (Cahill, 2018; Sharma et al., 2020). Furthermore, many countries have approved and are increasing over-the-counter access to OCs without age restrictions (ACOG, 2019; MHRA, 2021). Considering this and evidence from our analysis and other studies that OC use is associated with changes in brain functional connectivity, it is relevant for future studies to evaluate whether effects on brain functional connectivity is a mediator of OC use on mental health in adolescents.

Also, OC-related brain changes beyond brain network organization or mechanisms underlying such changes should be determined in future populations. Such changes may include key features of the serotonin signaling system (Larsen et al., 2020), since serotonin (5-HT) is a neurotransmitter implicated in modulating functional brain activity and neuropsychiatric pathophysiology (Roseman et al., 2014; Schaefer et al., 2014; Beliveau et al., 2015; Arnone et al., 2018) and estrogen and progesterone target the serotonin system (Barth et al., 2015).

### Summary

Our analyses have shown significant alterations in static and dynamic functional connectivity associated with OC use in an open access dataset of an individual scanned daily over 30 days two times 1-year apart. We show that the OC state had statistically significantly lower network modularity and characteristic path length in static estimates of network connectivity and changes in the dynamic connectivity. Due to the limitations of this study, we cannot conclude whether these phenomena generalize or a related to OC use *per se*. However, if the observed findings are related to OC use, this would point to OC-related effects on network dynamics in cognitive and emotional regulation networks. While it might stabilize cognitive and emotional function in some individuals, it might blunt it in others, contributing to an increased vulnerability toward psychiatric disorders. Although these findings are premature for clinical decision-making, they offer hypotheses to be tested in future cohorts designs to determine how the brain-network organization varies across the natural menstrual cycle and how it may be altered in the context of different OCs in different age groups. We propose that these analyses prompt further investigation into the effects of OCs on brain function, especially given the widespread use of these drugs in both adolescent and adult healthy individuals and those with psychiatric disturbances.

## Data Availability Statement

Publicly available datasets were analyzed in this study. This data can be found here: https://openneuro.org/datasets/ds002674/versions/1.0.5.

## Ethics Statement

The studies involving human participants were reviewed and approved by the University of California, Santa Barbara Human Subjects Committee. The participant provided their written informed consent to participate in this study.

## Author Contributions

KJ, DM, and AO wrote the initial draft of the manuscript. KJ, DM, AO, SL, and VF contributed to the conception and design of the study. DM, AO, and SB performed the statistical analyses. All authors contributed to the interpretation of the analyses, the manuscript revision, and have approved the submitted version.

## Conflict of Interest

DM’s salary was supported by an unrestricted grant from COMPASS Pathways Ltd., which had no involvement in this manuscript or related data collection. VF has received honorarium as a consultant for SAGE Therapeutics and Lundbeck Pharma A/S. The remaining authors declare that the research was conducted in the absence of any commercial or financial relationships that could be construed as a potential conflict of interest.

## Publisher’s Note

All claims expressed in this article are solely those of the authors and do not necessarily represent those of their affiliated organizations, or those of the publisher, the editors and the reviewers. Any product that may be evaluated in this article, or claim that may be made by its manufacturer, is not guaranteed or endorsed by the publisher.

## Footnotes

1
https://cran.r-project.org/

2
https://www.nitrc.org/projects/bnv/

## References

[B1] ACOG (2019). Over-the-Counter Access to Hormonal Contraception: ACOG Committee Opinion, Number 788. *Obstet. Gynecol.* 134 e96–e105. 31568364 10.1097/AOG.0000000000003473

[B2] Amoozegar F. Ronksley P. E. Sauve R. Menon B. K. (2015). Hormonal contraceptives and cerebral venous thrombosis risk: a systematic review and meta-analysis. *Front. Neurol.* 6:7. 10.3389/fneur.2015.00007 25699010 PMC4313700

[B3] Anderl C. Li G. Chen F. S. (2020). Oral contraceptive use in adolescence predicts lasting vulnerability to depression in adulthood. *J. Child Psychol. Psychiatry* 61 148–156. 10.1111/jcpp.13115 31461541

[B4] Anderl C. Wit A. E. Giltay E. J. Oldehinkel A. J. Chen F. S. (2021). Association between adolescent oral contraceptive use and future major depressive disorder: a prospective cohort study. *J. Child Psychol. Psychiatry* 63 333–341. 10.1111/jcpp.13476 34254301 PMC9291927

[B5] Arélin K. Mueller K. Barth C. Rekkas P. V. Kratzsch J. Burmann I. (2015). Progesterone mediates brain functional connectivity changes during the menstrual cycle—a pilot resting state MRI study. *Front. Neurosci.* 9:44. 10.3389/fnins.2015.00044 25755630 PMC4337344

[B6] Arnone D. Wise T. Walker C. Cowen P. J. Howes O. Selvaraj S. (2018). The effects of serotonin modulation on medial prefrontal connectivity strength and stability: a pharmacological fMRI study with citalopram. *Prog. Neuropsychopharmacol. Biol. Psychiatry* 84 152–159. 10.1016/j.pnpbp.2018.01.021 29409920 PMC5886357

[B7] Barth C. Steele C. J. Mueller K. Rekkas V. P. Arélin K. Pampel A. (2016). *In-vivo* dynamics of the human Hippocampus across the Menstrual Cycle. *Sci. Rep.* 6:32833. 10.1038/srep32833 27713470 PMC5054394

[B8] Barth C. Villringer A. Sacher J. (2015). Sex hormones affect neurotransmitters and shape the adult female brain during hormonal transition periods. *Front. Neurosci.* 9:37. 10.3389/fnins.2015.00037 25750611 PMC4335177

[B9] Bassett D. S. Bullmore E. T. (2016). Small-world brain networks revisited. *Neuroscientist* 23 499–516. 10.1177/1073858416667720 27655008 PMC5603984

[B10] Beliveau V. Svarer C. Frokjaer V. G. Knudsen G. M. Greve D. N. Fisher P. M. (2015). Functional connectivity of the dorsal and median raphe nuclei at rest. *Neuroimage* 116 187–195. 10.1016/j.neuroimage.2015.04.065 25963733 PMC4468016

[B11] Brønnick M. K. Økland I. Graugaard C. Brønnick K. K. (2020). The effects of hormonal contraceptives on the brain: a systematic review of neuroimaging studies. *Front. Psychol.* 11:556577. 10.3389/fpsyg.2020.556577 33224053 PMC7667464

[B12] Brynhildsen J. (2014). Combined hormonal contraceptives: prescribing patterns, compliance, and benefits versus risks. *Ther. Adv. Drug Saf.* 5 201–213. 10.1177/2042098614548857 25360241 PMC4212440

[B13] Bullmore E. Sporns O. (2012). The economy of brain network organization. *Nat. Rev. Neurosci.* 13 336–349. 10.1038/nrn3214 22498897

[B14] Cabral J. Vidaurre D. Marques P. Magalhães R. Moreira P. S. Soares J. M. (2017). Cognitive performance in healthy older adults relates to spontaneous switching between states of functional connectivity during rest. *Sci. Rep.* 7:5135. 10.1038/s41598-017-05425-7 28698644 PMC5506029

[B15] Cahill L. (2018). How does hormonal contraception affect the developing human adolescent brain? *Curr. Opin. Behav. Sci.* 23 131–135. 10.1016/j.cobeha.2018.06.015

[B16] Chan M. Y. Park D. C. Savalia N. K. Petersen S. E. Wig G. S. (2014). Decreased segregation of brain systems across the healthy adult lifespan. *Proc. Natl. Acad. Sci. U.S.A.* 111 E4997–E5006. 10.1073/pnas.1415122111 25368199 PMC4246293

[B17] Chang C. Glover G. H. (2009). Time-frequency dynamics of resting-state brain connectivity measured with fMRI. *Neuroimage* 50 81–98. 10.1016/j.neuroimage.2009.12.011 20006716 PMC2827259

[B18] Chu T. Li Y. Che K. Dong F. Ma H. Shi Y. (2021). Pregnancy leads to changes in the brain functional network: a connectome analysis. *Brain Imaging Behav.* 16 811–819. 10.1007/s11682-021-00561-1 34590214

[B19] Cohen J. R. D’Esposito M. (2016). The segregation and integration of distinct brain networks and their relationship to cognition. *J. Neurosci.* 36 12083–12094. 10.1523/jneurosci.2965-15.2016 27903719 PMC5148214

[B20] Dan R. Reuveni I. Canetti L. Weinstock M. Segman R. Goelman G. (2020). Trait-related changes in brain network topology in premenstrual dysphoric disorder. *Horm. Behav.* 124:104782. 10.1016/j.yhbeh.2020.104782 32470339

[B21] Dhillon I. S. Marcotte E. M. Roshan U. (2003). Diametrical clustering for identifying anti-correlated gene clusters. *Bioinformatics* 19 1612–1619. 10.1093/bioinformatics/btg209 12967956

[B22] Dubol M. Epperson C. N. Sacher J. Pletzer B. Derntl B. Lanzenberger R. (2020). Neuroimaging the menstrual cycle: a multimodal systematic review. *Front. Neuroendocrinol.* 60:100878. 10.1016/j.yfrne.2020.100878 33098847

[B23] Elliott M. L. Knodt A. R. Ireland D. Morris M. L. Poulton R. Ramrakha S. (2020). What is the test-retest reliability of common task-functional MRI measures? New empirical evidence and a meta-analysis. *Psychol. Sci.* 31 792–806. 10.1177/0956797620916786 32489141 PMC7370246

[B24] Esfahlani F. Z. Jo Y. Faskowitz J. Byrge L. Kennedy D. P. Sporns O. (2020). High-amplitude cofluctuations in cortical activity drive functional connectivity. *Proc. Natl. Acad. Sci. U.S.A.* 117 28393–28401. 10.1073/pnas.2005531117 33093200 PMC7668041

[B25] Filippi E. D. Uribe C. Avila-Varela D. S. Martínez-Molina N. Gashaj V. Pritschet L. (2021). The menstrual cycle modulates whole-brain turbulent dynamics. *Front. Neurosci.* 15:753820. 10.3389/fnins.2021.753820 34955718 PMC8695489

[B26] Frokjaer V. G. (2020). Pharmacological sex hormone manipulation as a risk model for depression. *J. Neurosci. Res.* 98 1283–1292. 10.1002/jnr.24632 32399989 PMC7383584

[B27] Green S. A. Graham B. M. (2022). Symptom fluctuation over the menstrual cycle in anxiety disorders, PTSD, and OCD: a systematic review. *Arch. Womens Ment. Health* 25 71–85. 10.1007/s00737-021-01187-4 34668073

[B28] Greenwell S. Faskowitz J. Pritschet L. Santander T. Jacobs E. G. Betzel R. F. (2021). High-amplitude network co-fluctuations linked to variation in hormone concentrations over menstrual cycle. *bioRxiv* [Preprint]. 10.1101/2021.07.29.453892 PMC10473261 37781152

[B29] Guen M. L. Schantz C. Régnier-Loilier A. Rochebrochard E. de L. (2021). Reasons for rejecting hormonal contraception in Western countries: a systematic review. *Soc. Sci. Med.* 284 114247. 10.1016/j.socscimed.2021.114247 34339927

[B30] Guo X. Yang F. Fan L. Gu Y. Ma J. Zhang J. (2021). Disruption of functional and structural networks in first-episode, drug-naïve adolescents with generalized anxiety disorder. *J. Affect. Disord.* 284 229–237. 10.1016/j.jad.2021.01.088 33618206

[B31] Iversen L. Fielding S. Lidegaard Ø. Hannaford P. C. (2020). Contemporary hormonal contraception and risk of endometrial cancer in women younger than age 50: a retrospective cohort study of Danish women. *Contraception* 102 152–158. 10.1016/j.contraception.2020.06.008 32592798

[B32] Keenan L. Kerr T. Duane M. Gundy K. V. (2018). Systematic review of hormonal contraception and risk of venous thrombosis. *Linacre Q.* 85 470–477. 10.1177/0024363918816683 32431379 PMC6322116

[B33] Kuehner C. Nayman S. (2021). Premenstrual exacerbations of mood disorders: findings and knowledge gaps. *Curr. Psychiatry Rep.* 23:78. 10.1007/s11920-021-01286-0 34626258 PMC8502143

[B34] Larsen S. V. Köhler-Forsberg K. Dam V. H. Poulsen A. S. Svarer C. Jensen P. S. (2020). Oral contraceptives and the serotonin 4 receptor: a molecular brain imaging study in healthy women. *Acta Psychiatr. Scand.* 142 294–306. 10.1111/acps.13211 33314049 PMC7586815

[B35] Levitt P. (2003). Structural and functional maturation of the developing primate brain. *J. Pediatr.* 143 35–45. 10.1067/s0022-3476(03)00400-1 14597912

[B36] Lewis C. A. Kimmig A.-C. S. Zsido R. G. Jank A. Derntl B. Sacher J. (2019). Effects of Hormonal contraceptives on mood: a focus on emotion recognition and reactivity, reward processing, and stress response. *Curr. Psychiatry Rep.* 21:115. 10.1007/s11920-019-1095-z 31701260 PMC6838021

[B37] MHRA (2021). *UK Medicines and Healthcare Products Regulatory Agency Press Release: First Progestogen-Only Contraceptive Pills to be Available to Purchase from Pharmacies.* Available online at: https://www.gov.uk/government/news/first-progesterone-only-contraceptive-pills-to-be-available-to-purchase-from-pharmacies (accessed December 28, 2021).

[B38] Montoya E. R. Bos P. A. (2017). How oral contraceptives impact social-emotional behavior and brain function. *Trends Cogn. Sci.* 21 125–136. 10.1016/j.tics.2016.11.005 28089524

[B39] Mueller J. M. Pritschet L. Santander T. Taylor C. M. Grafton S. T. Jacobs E. G. (2021). Dynamic community detection reveals transient reorganization of functional brain networks across a female menstrual cycle. *Netw. Neurosci.* 5 125–144. 10.1162/netn_a_00169 33688609 PMC7935041

[B40] Olsen A. S. Lykkebo-Valløe A. Ozenne B. Madsen M. K. Stenbæk D. S. Armand S. (2021). Psilocybin modulation of dynamic functional connectivity is associated with plasma psilocin and subjective effects. *medRxiv* [Preprint]. 10.1101/2021.12.17.21267992 36341951

[B41] Parkes A. Wight D. Henderson M. Stephenson J. Strange V. (2009). Contraceptive method at first sexual intercourse and subsequent pregnancy risk: findings from a secondary analysis of 16-Year-Old Girls from the RIPPLE and SHARE Studies. *J. Adolesc. Health* 44 55–63. 10.1016/j.jadohealth.2008.06.006 19101459 PMC2606907

[B42] Pinheiro J. Bates D. Sarkar S. D. R Core Team (2021). *nlme: Linear and Nonlinear Mixed Effects Models.* Available online at: https://CRAN.R-project.org/package=nlme.

[B43] Pinkerton J. V. Guico-Pabia C. J. Taylor H. S. (2010). Menstrual cycle-related exacerbation of disease. *Am. J. Obstet. Gynecol.* 202 221–231. 10.1016/j.ajog.2009.07.061 20207238 PMC3107848

[B44] Pletzer B. Crone J. S. Kronbichler M. Kerschbaum H. (2016). Menstrual cycle and hormonal contraceptive-dependent changes in intrinsic connectivity of resting-state brain networks correspond to behavioral changes due to hormonal status. *Brain Connect.* 6 572–585. 10.1089/brain.2015.0407 27239684

[B45] Pletzer B. Harris T.-A. Ortner T. (2017). Sex and menstrual cycle influences on three aspects of attention. *Physiol. Behav.* 179 384–390. 10.1016/j.physbeh.2017.07.012 28694156 PMC7115981

[B46] Pletzer B. Kronbichler M. Kerschbaum H. (2015). Differential effects of androgenic and anti-androgenic progestins on fusiform and frontal gray matter volume and face recognition performance. *Brain Res.* 1596 108–115. 10.1016/j.brainres.2014.11.025 25446458

[B47] Poromaa I. S. Segebladh B. (2012). Adverse mood symptoms with oral contraceptives. *Acta Obstet Gyn Scan* 91 420–427. 10.1111/j.1600-0412.2011.01333.x 22136510

[B48] Preti M. G. Bolton T. A. Ville D. V. D. (2016). The dynamic functional connectome: state-of-the-art and perspectives. *Neuroimage* 160 41–54. 10.1016/j.neuroimage.2016.12.061 28034766

[B49] Pritschet L. Santander T. Taylor C. M. Layher E. Yu S. Miller M. B. (2020). Functional reorganization of brain networks across the human menstrual cycle. *Neuroimage* 220:117091. 10.1016/j.neuroimage.2020.117091 32621974

[B50] Pritschet L. Taylor C. M. Santander T. Jacobs E. G. (2021b). Applying dense-sampling methods to reveal dynamic endocrine modulation of the nervous system. *Curr. Opin. Behav. Sci.* 40 72–78. 10.1016/j.cobeha.2021.01.012 35369044 PMC8975130

[B51] Pritschet L. Santander T. Taylor C. M. Layher E. Yu S. Miller M. B. (2021a). *28andMe.* Available online at: openneuro.org/datasets/ds002674/versions/1.0.5

[B52] Rapkin A. J. Korotkaya Y. Taylor K. C. (2019). Contraception counseling for women with premenstrual dysphoric disorder (PMDD): current perspectives. *Open Access J. Contracept.* 10 27–39. 10.2147/oajc.s183193 31572029 PMC6759213

[B53] Río J. P. D. Alliende M. I. Molina N. Serrano F. G. Molina S. Vigil P. (2018). Steroid hormones and their action in women’s brains: the importance of hormonal balance. *Front. Public Health* 6:141. 10.3389/fpubh.2018.00141 29876339 PMC5974145

[B54] Roach R. E. J. Helmerhorst F. M. Lijfering W. M. Stijnen T. Algra A. Dekkers O. M. (2015). Combined oral contraceptives: the risk of myocardial infarction and ischemic stroke. *Cochrane Database Syst. Rev.* 2015:CD011054. 10.1002/14651858.cd011054.pub2 26310586 PMC6494192

[B55] Rohatgi A. (2021). *Webplotdigitizer: Version 4.5.* Available online at: https://automeris.io/WebPlotDigitizer

[B56] Roseman L. Leech R. Feilding A. Nutt D. J. Carhart-Harris R. L. (2014). The effects of psilocybin and MDMA on between-network resting state functional connectivity in healthy volunteers. *Front. Hum. Neurosci.* 8:204. 10.3389/fnhum.2014.00204 24904346 PMC4034428

[B57] Rubinov M. Sporns O. (2009). Complex network measures of brain connectivity: uses and interpretations. *Neuroimage* 52 1059–1069. 10.1016/j.neuroimage.2009.10.003 19819337

[B58] Rubinov M. Sporns O. (2011). Weight-conserving characterization of complex functional brain networks. *Neuroimage* 56 2068–2079. 10.1016/j.neuroimage.2011.03.069 21459148

[B59] Schaefer A. Burmann I. Regenthal R. Arélin K. Barth C. Pampel A. (2014). Serotonergic modulation of intrinsic functional connectivity. *Curr. Biol.* 24 2314–2318. 10.1016/j.cub.2014.08.024 25242032

[B60] Schaefer A. Kong R. Gordon E. M. Laumann T. O. Zuo X.-N. Holmes A. J. (2018). Local-global parcellation of the human cerebral cortex from intrinsic functional connectivity MRI. *Cereb. Cortex* 28 3095–3114. 10.1093/cercor/bhx179 28981612 PMC6095216

[B61] Sharma R. Fang Z. Smith A. Ismail N. (2020). Oral contraceptive use, especially during puberty, alters resting state functional connectivity. *Horm. Behav.* 126:104849. 10.1016/j.yhbeh.2020.104849 32971138

[B62] Skovlund C. W. Mørch L. S. Kessing L. V. Lidegaard Ø (2016). Association of hormonal contraception with depression. *JAMA Psychiatry* 73 1154–1162. 10.1001/jamapsychiatry.2016.2387 27680324

[B63] Sporns O. Betzel R. F. (2016). Modular brain networks. *Annu. Rev. Psychol.* 67 613–640. 10.1146/annurev-psych-122414-033634 26393868 PMC4782188

[B64] Taylor C. M. Pritschet L. Olsen R. K. Layher E. Santander T. Grafton S. T. (2020). Progesterone shapes medial temporal lobe volume across the human menstrual cycle. *Neuroimage* 220:117125. 10.1016/j.neuroimage.2020.117125 32634592

[B65] Tozzi L. Zhang X. Chesnut M. Holt-Gosselin B. Ramirez C. A. Williams L. M. (2021). Reduced functional connectivity of default mode network subsystems in depression: meta-analytic evidence and relationship with trait rumination. *Neuroimage Clin.* 30:102570. 10.1016/j.nicl.2021.102570 33540370 PMC7856327

[B66] Wickham H. (2016). *ggplot2: Elegant Graphics for Data Analysis.* Available online at: https://ggplot2.tidyverse.org

[B67] Xia M. Wang J. He Y. (2013). BrainNet viewer: a network visualization tool for human brain connectomics. *PLoS One* 8:e68910. 10.1371/journal.pone.0068910 23861951 PMC3701683

[B68] Xu S. Deng W. Qu Y. Lai W. Huang T. Rong H. (2021). The integrated understanding of structural and functional connectomes in depression: a multimodal meta-analysis of graph metrics. *J. Affect. Disord.* 295 759–770. 10.1016/j.jad.2021.08.120 34517250

[B69] Yan C.-G. Chen X. Li L. Castellanos F. X. Bai T.-J. Bo Q.-J. (2019). Reduced default mode network functional connectivity in patients with recurrent major depressive disorder. *Proc. Natl. Acad. Sci. U.S.A.* 116 9078–9083. 10.1073/pnas.1900390116 30979801 PMC6500168

[B70] Yeo B. T. T. Krienen F. M. Sepulcre J. Sabuncu M. R. Lashkari D. Hollinshead M. (2011). The organization of the human cerebral cortex estimated by intrinsic functional connectivity. *J. Neurophysiol.* 106 1125–1165. 10.1152/jn.00338.2011 21653723 PMC3174820

[B71] Yun J.-Y. Kim Y.-K. (2021). Graph theory approach for the structural-functional brain connectome of depression. *Prog. Neuropsychopharmacol. Biol. Psychiatry* 111:110401. 10.1016/j.pnpbp.2021.110401 34265367

[B72] Zettermark S. Vicente R. P. Merlo J. (2018). Hormonal contraception increases the risk of psychotropic drug use in adolescent girls but not in adults: a pharmacoepidemiological study on 800 000 Swedish women. *PLoS One* 13:e0194773. 10.1371/journal.pone.0194773 29566064 PMC5864056

[B73] Zhang Y. Liu X. Hou Z. Yin Y. Xie C. Zhang H. (2021). Global topology alteration of the brain functional network affects the 8-week antidepressant response in major depressive disorder. *J. Affect. Disord.* 294 491–496. 10.1016/j.jad.2021.07.078 34330044

