# Supplementary material for: Effects of an Oral Contraceptive on Dynamic Brain States and Network Modularity in a Serial Single-Subject Study
Source: Front Neurosci. 2022 Jun 14;16:855582. doi: 10.3389/fnins.2022.855582 (PMC9237452; doi:10.3389/fnins.2022.855582)
Supplement: Supplementary file 1 [file Data_Sheet_1.pdf]

Supplementary material for

Effects of an Oral Contraceptive on Dynamic Brain States and Network Modularity in a Serial Single-Subject Study

1 Supplementary Figures

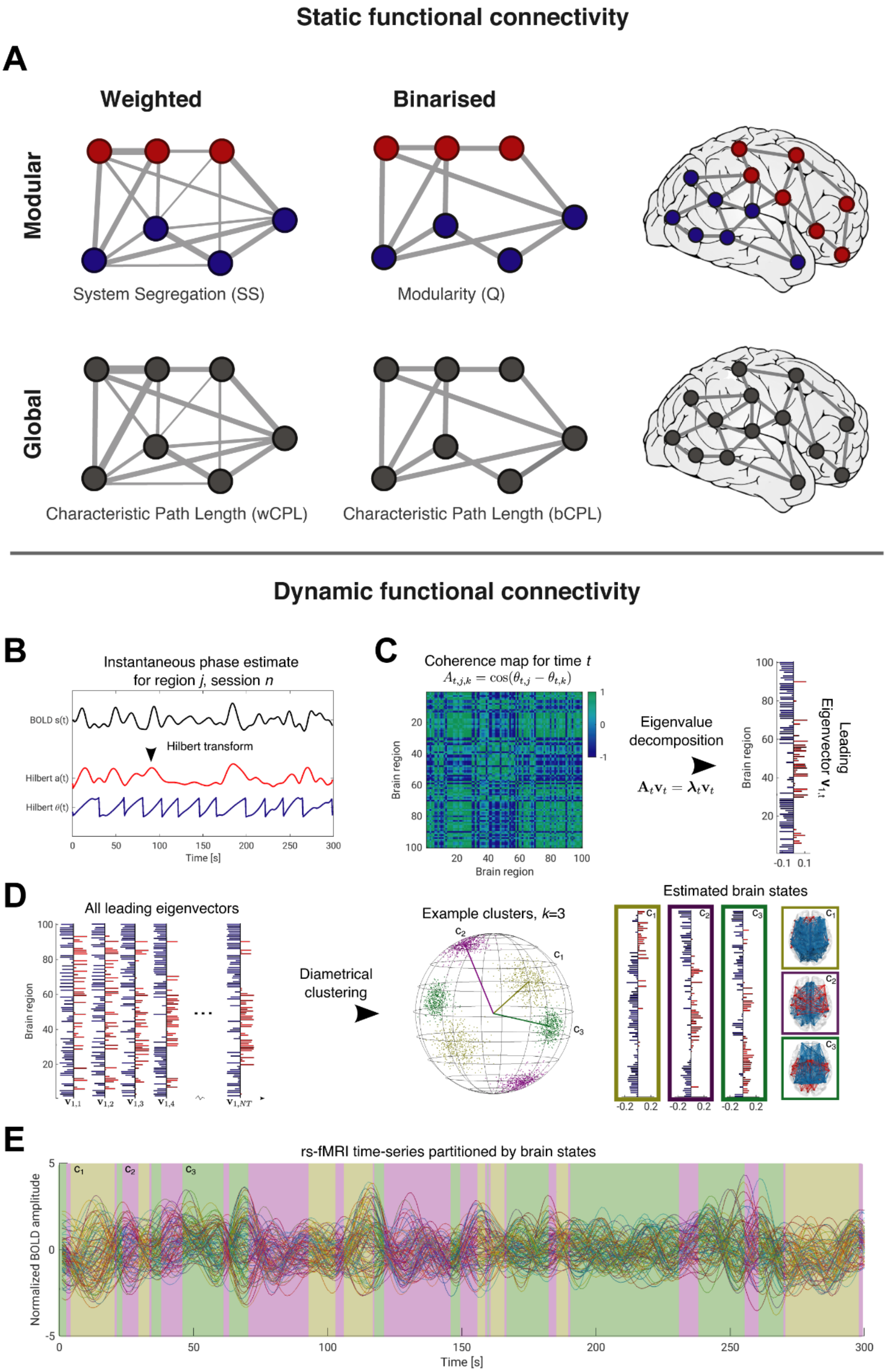

Supplementary Figure S1: Graph theory static connectivity and Leading Eigenvector Dynamics Analysis (LEiDA) and diametrical clustering measurement of dynamic connectivity

(A) System segregation and modularity describe the functional subdivision of the brain into discrete

subnetworks, while characteristic path length describes the capacity for information flow across nodes (brain areas). In weighted networks edges are represented as continuous values, each one defining the edge strength; whereas in binarised networks, edges below a threshold are pruned, retaining only edges above a threshold. In LEiDA, (B) the Hilbert transform is used to separate amplitude a(t) and phase $\boldsymbol{\theta(t)}$ information from each regional rs-fMRI time series, for each scan session. (C) For each time-point, *t*, a coherence map is estimated and decomposed using the eigenvalue decomposition, retaining the leading eigenvector for further analysis. (D) The set of leading eigenvectors within and across scan sessions is clustered into *k* predefined clusters using diametrical clustering, e.g., three clusters c1-c3 shown here. (E) The original rs-fMRI time-series is partitioned according to the estimated brain states; fractional occurrence, i.e., the fraction of time points during which the brain is in a given brain state, can be calculated.

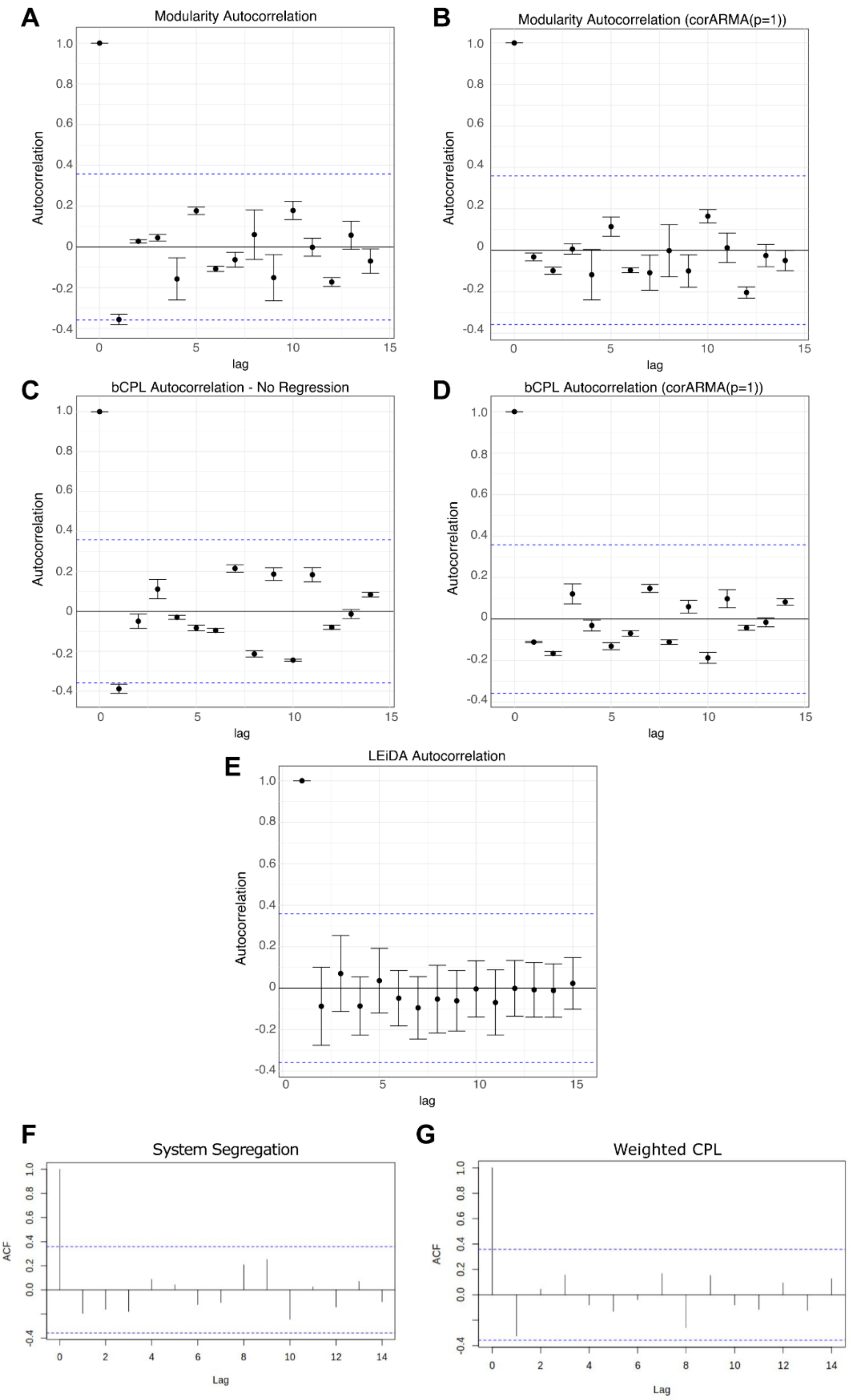

Supplementary Figure S2: Autocorrelation of (A-B) Modularity, (C-D) Binarized Characteristic Path Length, (D) LEiDA, (E) System Segregation, and (F) Weighted Characteristic Path Length. For LEiDA, we estimated the autocorrelation across all 209 estimated brain states (for $k \in \{2, \ldots, 20\}$) and report the average and standard deviation of the coefficients (E).

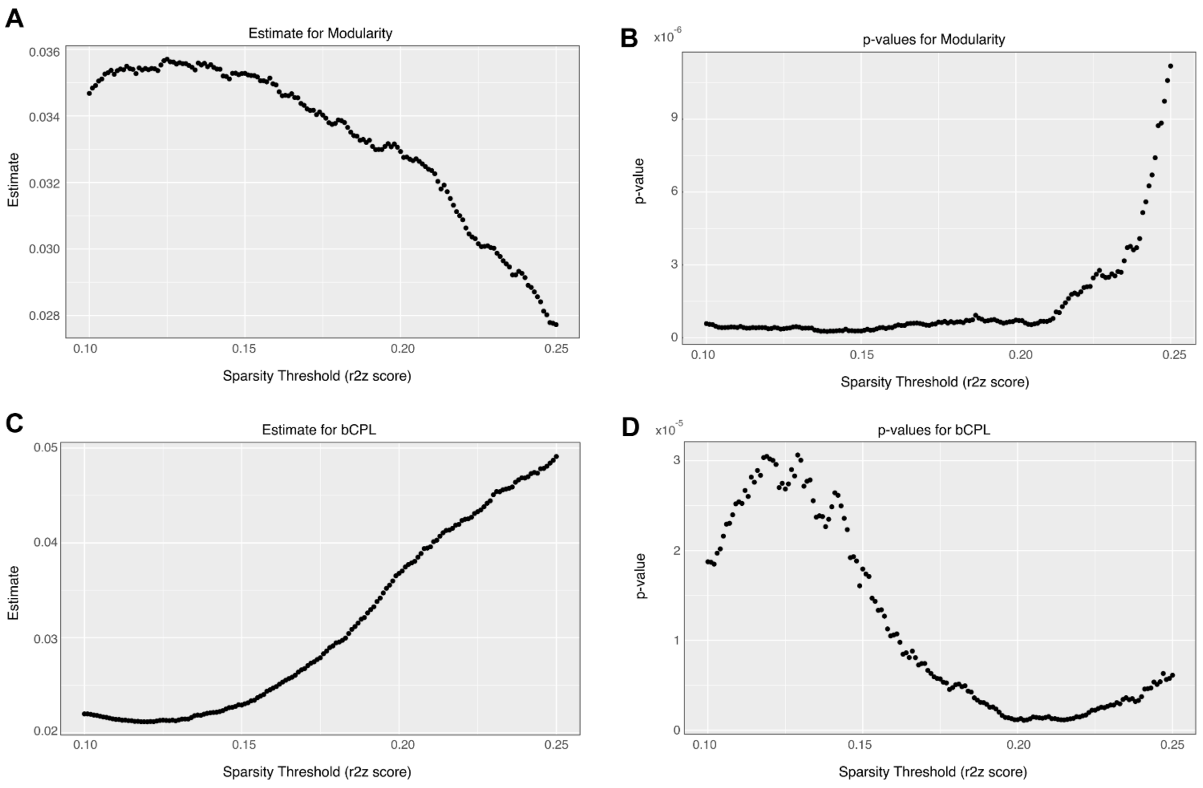

Supplementary Figure S3: Modularity and bCPL estimates across a range of sparsity thresholds. Both modularity and bCPL are sensitive to the threshold r2z-score as a graph pruning parameter. Here, paired t-test estimates for modularity (A) and corresponding p-values (B) indicate a statistically significant difference between OC and naturally cycling scans across the range of thresholds. Note that the significance threshold of 0.05 is not shown in figures B and D, as this would make the points appear as straight lines.

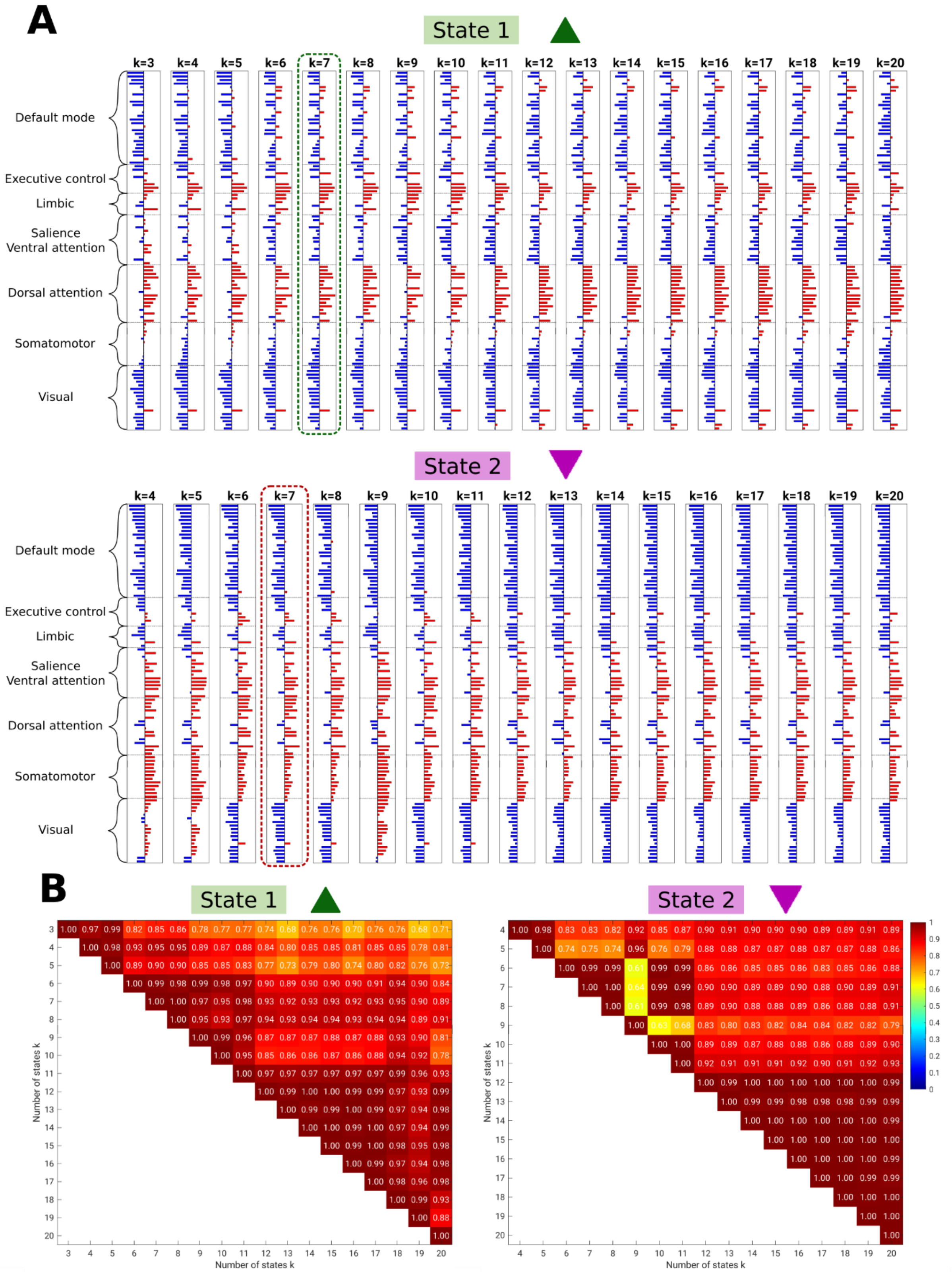

Supplementary Figure S4: Highlighted centroids across *k* including structural similarity (A) and between-*k* Pearson correlation (B).

2 Supplementary text

2.1 MRI acquisition

The subject underwent a daily MRI scan session on a Siemens 3T Prisma scanner equipped with a 64-channel, phased-array head coil. High-resolution T1-weighted images were acquired using a magnetisation prepared rapid gradient echo (MPRAGE) sequence (TR = 2500 ms, TE = 2.31 ms, TI = 934 ms, flip angle = 7°, 0.8 mm slice thickness) followed by a gradient echo field map (TR = 758 ms, TE1 = 4.92 ms, TE2 = 7.38 ms, flip angle = 60°).

The subsequent rs-fMRI scan lasted 10 min and was acquired with a T2∗-weighted multiband echo-planar imaging (EPI) sequence sensitive to the blood oxygenation level-dependent (BOLD) contrast (72 oblique slices, TR = 720 ms, TE = 37 ms, voxel size = 2x2x2 mm, flip angle = 52°, multiband factor = 8). A custom headcase minimised movement, and the motion was deemed negligible, with less than 130 μm framewise displacement on average each day (Taylor et al., 2020).

2.2 fMRI Preprocessing

Each of the acquired 10-minute rs-fMRI scan sessions were preprocessed separately in SPM12 (http://www.fil.ion.ucl.ac.uk/spm). The pipeline included 1) calculation of voxel displacement map, 2) spatial realignment and bias field unwarping, 3) co-registration of the structural T1-weighted image to the first functional volume of the scan session, 4) normalisation of the coregistered anatomical volume to MNI152-space and subsequent transformation of the functional volumes using the estimated transformation matrix, 5) smoothing of normalised functional images (6 mm FWHM Gaussian kernel), and 6) segmentation of the normalised T1-weighted image into grey matter, white matter, and cerebrospinal fluid (CSF) maps. Functional images were temporally denoised using CONN (Whitfield-Gabrieli and Nieto-Castanon, 2012), including voxel-wise linear detrending and nuisance regression of 1) three translation and three rotation parameters from the spatial realignment step and their first-order derivates, and 2) anatomical component correction (aCompCor) (Behzadi et al., 2007) based on the first five principal components from white matter and CSF time-series and their first derivatives. Subsequently, time-series data were band-pass filtered voxel between 0.008 and 0.09 Hz. The 100- and 400-region Schaefer atlases (Schaefer et al., 2018) labelled according to seven canonical resting-state networks (Yeo et al., 2011) were used to extract denoised regional time series for further analysis.

2.3 Hilbert transform

Regional instantaneous BOLD-phases were estimated using the Hilbert transform

$$s_h(t) = s(t) * \frac{1}{\pi t},$$

where $*$ represents the convolution operator, to create the complex analytic signal $z(t) = s(t) + is_h(t)$. The analytic signal encompasses both signal amplitude and oscillatory information by projecting it onto counterclockwise motion, phase-shifted by $\frac{\pi}{4}$. The instantaneous phases may then be found as the point-wise angle to the real axis

$$\theta(t) = \arctan\left(\frac{s_h(t)}{s(t)}\right),$$

and represent the oscillatory dynamics of the BOLD signal bounded by $\theta \in [-\pi; \pi)$. Similarly, the instantaneous amplitude of the BOLD signal may be found as the modulus of the analytic signal, however, in the attempt to uncover phase coherence networks, amplitude information is disregarded here.

2.4 Graph-theory measures

2.4.1 System Segregation

System segregation (SS) measures the relative strength of within-network connectivity compared to between-network connectivity (Chan et al., 2014), and is defined as

$$\boldsymbol{SS} = \frac{\bar{\boldsymbol{z}}_{\boldsymbol{w}} - \bar{\boldsymbol{z}}_{\boldsymbol{b}}}{\bar{\boldsymbol{z}}_{\boldsymbol{w}}},$$

where $\bar{z}_w$ (within-network) represents the average r2z score for all edges that connect two nodes from the same network, and $\bar{z}_b$ (between-network) represents the average r2z score for all edges that connect two nodes from distinct networks. Conceptually, higher system segregation implies greater within- relative to between-network connectivity strength.

2.4.2 Characteristic Path Length

Characteristic Path Length (CPL), is the average shortest path length between all pairs of nodes; it is sometimes referred to as "average path length" and is the inverse of "global efficiency" (Rubinov and Sporns, 2009). CPL is a global measure of the functional integration of a system (Watts and Strogatz, 1998). Low values indicate short paths between nodes, putatively representing a highly integrated system. CPL can be described using either a weighted or binarised connectivity matrix (wCPL or bCPL, respectively). The CPL for node *i* is defined as:

$$\bar{d}_i = \frac{1}{n-1} \sum_{j=1}^{n} d_{ij},$$

where $\bar{d}_i$ represents the average path length from node *i* to all other nodes *j* (*n* = 400, being the total number of nodes) and d_{ij} represents the shortest path length between nodes *i* and *j*. CPL is then the average across *n* nodes. The shortest path between any given pair of nodes is the path that minimises the total length between a given pair of nodes, which may be direct from node *i* to node *j*, or via other nodes, in which case the path length is the sum of the lengths between each node. For wCPL individual edge lengths are defined as 1-*r* where *r* is the connectivity strength expressed as the Pearson's correlation coefficient (Rubinov and Sporns, 2009). bCPL was calculated as wCPL except using the binarised connectivity matrix for each scan session; individual edge lengths between nodes were either 1 for super-threshold edges or infinite for sub-threshold edges.

The graph-theoretical interpretation of negatively weighted connectivity is not well established (Rubinov and Sporns, 2011) and thus connectivity values are set to zero as recommended in (Rubinov and Sporns, 2011), resulting in a path length of 1.

2.4.3 Modularity

Modularity (Q) is a measure of the number of suprathreshold, within-network connections relative to the number of suprathreshold, between-network connections (Cohen and D'Esposito, 2016; Sporns and Betzel, 2016) and is defined as

$$Q = \sum_{i=1}^{m}(e_{ii} - a_i^2),$$

where e_{ii} represents the fraction of within-network connections that are suprathreshold for network i, a_i represents the fraction of between-network connections where one node belongs to network *i* that are suprathreshold, while *m* represents the total number of networks (i.e., seven). As such, modularity is a measure of the degree to which a system is segregated into functionally distinct networks as opposed to homogenous connectivity across the system. Higher values indicate greater segregation into distinct networks.

2.5 Leading Eigenvector Dynamics Analysis

We estimate dynamic connectivity structures using LEiDA (Cabral et al., 2017), followed by diametrical clustering as in (Olsen et al., 2021)(see Figure S1B-E). The brain was parcellated into cortical regions from the Schaefer-100 atlas (Schaefer et al., 2018). Regional instantaneous BOLD-phases were estimated using the Hilbert transform (see section 2.3), which separates instantaneous phases representing the oscillatory dynamics of the BOLD signal from its amplitude.

Interregional synchrony is summarised for each time point t, for each scan session, in the diagonally symmetrical phase coherence map $\boldsymbol{A}_t$ with elements

$$A_{t,j,k} = \cos(\theta_{t,j} - \theta_{t,k})$$

for regions j and k. As per LEiDA, the first eigenvector, $\boldsymbol{v}_{1,t}$, following an eigenvalue decomposition of the brain-wide coherence map is retained, thereby capturing the dominant instantaneous connectivity pattern.

2.5.1 Diametrical clustering

Eigenvectors are ambiguous in both their norm and sign and are therefore well-clustered with respect to an antipodally symmetric unit hypersphere (Watson, 1965; Sra and Karp, 2013; Olsen et al., 2021). Diametrical clustering serves this purpose, (Dhillon et al., 2003; Sra and Karp, 2013) and is essentially a modified *k*-means procedure, where distance $d_{t,c}$ is measured for every time point *t* using the squared Pearson correlation, e.g.,

$$d_{t,c} = \left(\boldsymbol{v}_{1,t}^T \boldsymbol{\mu}_c\right)^2,$$

where $\boldsymbol{\mu}_c$ is the centroid of cluster c. We group all leading eigenvectors concatenated across scan sessions into *k* clusters (i.e., brain states). Diametrical clustering is initialised using an implementation of *k*-means++ (Arthur and Vassilvitskii, 2007). The optimal *k* is not well-defined; therefore, we ran our model for *k* ranging from 2 to 20, in line with previous studies examining brain dynamics using LEiDA (Cabral et al., 2017; Figueroa et al., 2019; Kringelbach et al., 2020; Olsen et al., 2021). For each *k*, the best of 5 trained models, in terms of average distance of points to the nearest centroid, was selected. We define brain state fractional occurrence as the fraction of volumes within a single rs-fMRI scan session assigned to a specific brain state. This produces $N = 60$ fractional occurrence estimates per brain state.

2.6 Atlas considerations

In this study, we investigated graph-theoretical measures of brain activity and dynamic functional connectivity using the Schaefer atlases parcellated into 400 and 100 regions, respectively (Schaefer et al., 2018). The original article suggested the use of either the 400 or the 1000-region parcellations for the analysis of functional data. However, clustering methods, including diametrical clustering, suffer from the "curse of dimensionality", hence a lower number of regions is preferable. We have previously analysed a similar-sized dataset using a 90-dimensional atlas and observed reasonable convergence properties (Olsen et al., 2021). Regions in the Schaefer atlas are labelled according to a

canonical functional network as defined in (Yeo et al., 2011), thereby enabling the interpretation of dFC-results in terms of network implication. As such, the Schaefer atlas is preferable over anatomical atlases where regions are not easily assigned to groups. In contrast, region-specific alterations are easily overlooked when working with network atlases, and similarly, in this study, we did not focus on anatomically localised subnetworks.

3 References

Arthur, D., and Vassilvitskii, S. (2007). K-Means++: The Advantages of Careful Seeding. in *Proceedings of the 18th Annual ACM-SIAM Symposium on Discrete Alogrithms (SODA 2007)* (New Orleans, Louisiana, USA), 1027–1035. doi: 10.1145/1283383.1283494.

Behzadi, Y., Restom, K., Liau, J., and Liu, T. T. (2007). A component based noise correction method (CompCor) for BOLD and perfusion based fMRI. *Neuroimage* 37, 90–101. doi: 10.1016/j.neuroimage.2007.04.042.

Cabral, J., Vidaurre, D., Marques, P., Magalhães, R., Moreira, P. S., Soares, J. M., et al. (2017). Cognitive performance in healthy older adults relates to spontaneous switching between states of functional connectivity during rest. *Sci Rep-uk* 7, 5135. doi: 10.1038/s41598-017-05425-7.

Chan, M. Y., Park, D. C., Savalia, N. K., Petersen, S. E., and Wig, G. S. (2014). Decreased segregation of brain systems across the healthy adult lifespan. *P Natl Acad Sci Usa* 111, E4997-5006. doi: 10.1073/pnas.1415122111.

Cohen, J. R., and D'Esposito, M. (2016). The Segregation and Integration of Distinct Brain Networks and Their Relationship to Cognition. *J Neurosci* 36, 12083–12094. doi: 10.1523/jneurosci.2965-15.2016.

Dhillon, I. S., Marcotte, E. M., and Roshan, U. (2003). Diametrical clustering for identifying anti-correlated gene clusters. *Bioinformatics* 19, 1612–1619. doi: 10.1093/bioinformatics/btg209.

Figueroa, C. A., Cabral, J., Mocking, R. J. T., Rapuano, K. M., Hartevelt, T. J. van, Deco, G., et al. (2019). Altered ability to access a clinically relevant control network in patients remitted from major depressive disorder. *Hum Brain Mapp* 40, 2771–2786. doi: 10.1002/hbm.24559.

Kringelbach, M. L., Cruzat, J., Cabral, J., Knudsen, G. M., Carhart-Harris, R., Whybrow, P. C., et al. (2020). Dynamic coupling of whole-brain neuronal and neurotransmitter systems. *P Natl Acad Sci Usa*, 201921475. doi: 10.1073/pnas.1921475117.

Olsen, A. S., Lykkebo-Valløe, A., Ozenne, B., Madsen, M. K., Stenbæk, D. S., Armand, S., et al. (2021). Psilocybin modulation of dynamic functional connectivity is associated with plasma psilocin and subjective effects. *Medrxiv*, 2021.12.17.21267992. doi: 10.1101/2021.12.17.21267992.

Rubinov, M., and Sporns, O. (2009). Complex network measures of brain connectivity: uses and interpretations. *Neuroimage* 52, 1059–69. doi: 10.1016/j.neuroimage.2009.10.003.

Rubinov, M., and Sporns, O. (2011). Weight-conserving characterization of complex functional brain networks. *Neuroimage* 56, 2068–2079. doi: 10.1016/j.neuroimage.2011.03.069.

Schaefer, A., Kong, R., Gordon, E. M., Laumann, T. O., Zuo, X.-N., Holmes, A. J., et al. (2018). Local-Global Parcellation of the Human Cerebral Cortex from Intrinsic Functional Connectivity MRI. *Cereb Cortex New York N Y 1991* 28, 3095–3114. doi: 10.1093/cercor/bhx179.

Sporns, O., and Betzel, R. F. (2016). Modular Brain Networks. *Annu Rev Psychol* 67, 613–640. doi: 10.1146/annurev-psych-122414-033634.

Sra, S., and Karp, D. (2013). The multivariate Watson distribution: Maximum-likelihood estimation and other aspects. *J Multivariate Anal* 114, 256–269. doi: 10.1016/j.jmva.2012.08.010.

Taylor, C. M., Pritschet, L., Olsen, R. K., Layher, E., Santander, T., Grafton, S. T., et al. (2020). Progesterone shapes medial temporal lobe volume across the human menstrual cycle. *Neuroimage* 220, 117125. doi: 10.1016/j.neuroimage.2020.117125.

Watson, G. S. (1965). Equatorial Distributions on a Sphere. *Biometrika* 52, 193. doi: 10.2307/2333824.

Watts, D. J., and Strogatz, S. H. (1998). Collective dynamics of 'small-world' networks. *Nature* 393, 440–442. doi: 10.1038/30918.

Whitfield-Gabrieli, S., and Nieto-Castanon, A. (2012). Conn : A Functional Connectivity Toolbox for Correlated and Anticorrelated Brain Networks. *Brain Connectivity* 2, 125–141. doi: 10.1089/brain.2012.0073.

Yeo, B. T. T., Krienen, F. M., Sepulcre, J., Sabuncu, M. R., Lashkari, D., Hollinshead, M., et al. (2011). The organization of the human cerebral cortex estimated by intrinsic functional connectivity. *J Neurophysiol* 106, 1125–65. doi: 10.1152/jn.00338.2011.
